# The effect of behaviour change interventions on changes in physical activity and anthropometrics in ambulatory hospital settings: a systematic review and meta-analysis

**DOI:** 10.1186/s12966-020-01076-6

**Published:** 2021-01-07

**Authors:** Stephen Barrett, Stephen Begg, Paul O’Halloran, Owen Howlett, Jack Lawrence, Michael Kingsley

**Affiliations:** 1 La Trobe Rural Health School, La Trobe University, PO Box 199, Bendigo, Victoria 3552 Australia; 2 Health Promotion Department, Bendigo Health Care Group, PO Box 126, Bendigo, Victoria 3552 Australia; 3 School of Psychology and Public Health, La Trobe University, Bundoora, Victoria 3068 Australia; 4 Research and Innovation, Bendigo Health Care Group, PO Box 126, Bendigo, Victoria 3552 Australia; 5 Gurri Wanyarra Welllbing Centre, Bendigo, Victoria 3550 Australia; 6 Holsworth Research Initiative, La Trobe Rural Health School, La Trobe University, PO Box 199, Bendigo, Victoria 3552 Australia; 7 Department of Exercise Sciences, University of Auckland, Newmarket, 1023 New Zealand

## Abstract

**Background:**

The aim of this systematic review and meta-analysis was to investigate whether behaviour change interventions promote changes in physical activity and anthropometrics (body mass, body mass index and waist circumference) in ambulatory hospital populations.

**Methods:**

Randomised controlled trials were collected from five bibliographic databases (MEDLINE, Embase, CINAHL, The Cochrane Central Register of Controlled Trials (CENTRAL) and PsycINFO). Meta-analyses were conducted using change scores from baseline to determine mean differences (MD), standardised mean differences (SMD) and 95% confidence intervals (95% CI). The Grades of Recommendation, Assessment, Development and Evaluation approach was used to evaluate the quality of the evidence.

**Results:**

A total of 29 studies met the eligibility criteria and 21 were included in meta-analyses. Behaviour change interventions significantly increased physical activity (SMD: 1.30; 95% CI: 0.53 to 2.07, *p* < 0.01), and resulted in significant reductions in body mass (MD: -2.74; 95% CI: − 4.42 to − 1.07, *p* < 0.01), body mass index (MD: -0.99; 95% CI: − 1.48 to − 0.50, *p* < 0.01) and waist circumference (MD: -2.21; 95% CI: − 4.01 to − 0.42, *p* = 0.02). The GRADE assessment indicated that the evidence is very uncertain about the effect of behaviour change interventions on changes in physical activity and anthropometrics in ambulatory hospital patients.

**Conclusions:**

Behaviour change interventions initiated in the ambulatory hospital setting significantly increased physical activity and significantly reduced body mass, body mass index and waist circumference. Increased clarity in interventions definitions and assessments of treatment fidelity are factors that need attention in future research.

PROSPERO registration number: CRD42020172140.

## Background

Chronic diseases are leading causes of ill health worldwide [1]. Modifiable risk factors such as insufficient physical activity (PA), poor diet, and obesity are associated with an increased risk for chronic disease [2] and, due to medical improvements, individuals are living with chronic disease for longer periods [3, 4]. Increased survival amongst people with chronic disease results in a higher prevalence of morbidity, and lower quality of life [4]. As a result, secondary prevention has become important for chronic disease management globally [5].

Secondary prevention aims to reduce the impact of chronic disease through early detection and treatment. Behaviour change as a secondary prevention strategy is emerging as a way to mitigate the impact of disease and slow down disease progression [6]. Hospitals are important settings for the delivery of secondary prevention programs given their unique access to members of the local community who might benefit [7]. Hospital attendees are not necessarily registered with a GP and may not be actively engaged with community health promotion services [7], but because their health is already compromised, these individuals can be readily motivated to engage with lifestyle behaviour changes [8]. Behaviour change interventions are advocated as the first-line approach to behavioural risk factor management [9].

Results from recent meta-analyses indicate that secondary prevention behaviour change interventions result in positive effects in PA [10, 11], anthropometrics [11] and cardiovascular health [12]. These reviews included studies from hospital settings, though many studies recruited patients from the inpatient setting [10, 11]. Contextual differences exist in recruiting individuals for behaviour change interventions from the admitted versus ambulatory hospital setting [13, 14].

In the inpatient setting, patients are removed from their home environments, often suffering from a serious condition, and are potentially confined to their bed or the hospital room [14]. Being hospitalised has been identified as a major life event, increasing the likelihood of engaging in recommended care [15]. The inpatient environment imposes unique constraints on individuals, including their perception of autonomy of their care [13]. Consequently, the decision to initiate health behaviour change is potentially impacted by the inpatient setting [13].

Ambulatory hospital patients, on the other hand, engage in care under different circumstances. These individuals are community-dwelling, and maintain more autonomy over their care, including decisions regarding the treatment plan, or when they can expect to see the doctor next [16]. The delivery of preventive health care in the ambulatory hospital setting should be targeted, patient-centred, and characterised by interventions that support people with chronic disease risk factors and should include self-management support wherever possible [17]. Knowledge of the impact of behaviour change interventions on ambulatory hospital patients might allow prioritising preventive interventions in the ambulatory hospital setting for the prevention and management of chronic disease. To the best of our knowledge, no review has examined the effect of behaviour change interventions that address changes in PA and anthropometrics in non-admitted secondary care patients. Therefore, the aim of this review was to examine the effect of behaviour change interventions on changes and maintenance on PA, and anthropometrics, initiated in the ambulatory hospital setting only.

### Research question

Do behaviour change interventions result in positive changes and maintenance in PA and anthropometrics in adults attending ambulatory hospital clinics?

## Methods

A systematic review and meta-analysis was conducted according to the Preferred Reporting Items for Systematic Reviews and Meta-Analyses (PRISMA) [18] (Additional file 1). This review was registered with PROSPERO (registration ID: CRD42020172140).

### Data sources and search strategies

To avoid duplication, a search was undertaken in the Cochrane Database of Systematic Reviews, PubMed Clinical Queries and PROSPERO International prospective register of systematic reviews to confirm that no similar systematic reviews or protocols have been conducted. Eligible studies were collected (from inception until May 2020) using computer-based searches in MEDLINE, Embase, CINAHL, Web of Science, PsycINFO and The Cochrane Central Register of Controlled Trials (CENTRAL) electronic databases. Database-specific search strategies were developed with the guidance of professional clinical librarians. The database searches were performed using three main concepts: ambulatory secondary hospital care, lifestyle behaviour change interventions and outcomes (PA and anthropometric measures). For each main concept relevant related terms and keywords were included in the sensitive search (search details for MEDLINE are presented in Additional file 2).

Two additional steps were undertaken to ensure the comprehensiveness of our search. Firstly, searches were undertaken in clinical trial registries, including ClinicalTrials.gov, EU Clinical Trials Register, Australian New Zealand Clinical Trials Registry and the World Health Organization International Clinical Trial Registry Platform to source relevant ongoing and unpublished trials. Secondly, we performed a snowball search on reference lists, and grey literature databases.

### Eligibility criteria

The term behaviour change interventions is used to define coordinated activities designed to change specified behaviour outcomes [19]. For the purpose of this review, we included behaviour change interventions that specifically aimed to elicit changes in anthropometrics and/or PA changes through the use of behaviour modification components and strategies. Inclusion criteria to select studies were: 1) Study population: adult (aged 18 or older) ambulatory hospital patients; 2) Types of studies: peer-reviewed randomised controlled trials regarding a behaviour change intervention compared to a control intervention or usual care comparison group. The behaviour change intervention could be a single intervention or a multi-component intervention, but needed to include at least one session that was delivered in a 1:1 format (delivered in person, via the phone or telehealth) because of the importance of an individualised approach to self-management [20]; 3) Primary outcomes: PA, anthropometric measures – body mass, body mass index (BMI) and waist circumference (WC). Due to the clinical relevance of changes in body mass, BMI and WC, an a priori decision was made to undertake an meta-analysis on each outcome individually [21, 22]. Behavioural science highlights the need to draw the distinction between initial behaviour change and behaviour change maintenance [23]. To establish the maintenance effect of interventions, studies that included a follow-up duration of less than 12 weeks were excluded.

Studies were included that reported any of the following physical activity outcome measures: changes in daily steps, METs per week (METs/wk) or minutes per day/week of moderate to vigorous physical activity (MVPA) measured subjectively (e.g., self-report) or objectively at baseline and post intervention.

### Study selection

Studies were entered into Review Manager (Version 5.3; The Cochrane Collaboration, Denmark) and duplicates were removed. Screening was carried out using Covidence (Covidence Systematic Review Software, Veritas Health Innovation, Melbourne, Australia). Two authors independently screened title/abstracts and full text. Studies were systematically excluded when they did not meet the pre-specified inclusion criteria. Disagreements between reviewers were resolved by discussion, or where required with consensus of a third reviewer.

### Data extraction

Data were independently extracted by two reviewers. Data extraction was performed with the aid of a predesigned and piloted data collection form. For each study, the reviewers extracted information with respect to study characteristics (type of study, population description, focused disease or condition); study participants (sample size, demographics); methods (intervention duration, type and frequency, fidelity blinding, amount of intervention groups, number of included participants, the number of individuals that were randomised and analysed); the professional background of the person delivering the intervention); and outcome variables (outcome definition, unit of measurement, time points measured and reported). Continuous data including, means, standard deviations and the sample size numbers were extracted. When information was unclear, insufficient or missing, the authors of trials were contacted for clarifications and additional results. Where standard deviations were not available, measures of variance were estimated from the standard error of a mean, confidence intervals or *p*-values according to the Cochrane Handbook for Systematic Reviews of Interventions the Cochrane Collaboration [19]. When data were presented as median and interquartile range, the mean and standard deviation were estimated using the formula from Hozo et al. [24].

### Study quality assessment

The risk of bias of the included studies was assessed by two reviewers independently using the Cochrane Risk of Bias assessment tool [25]. The following methodological criteria were assessed: sequence generation; allocation concealment; blinding of participants, personnel and outcome assessors; incomplete outcome data; selective outcome reporting; and other potential threats to validity [25]. Each of these criteria were judged and classified as ‘low risk’, ‘high risk’ or ‘unclear risk’ of bias.

The overall strength of the evidence was assessed using the Grading of Recommendations, Assessment, Development and Evaluation (GRADE) [26] system through the GRADEpro 3.6 software (GRADEpro GDT: GRADEpro Guideline Development Tool [Software]; McMaster University, USA). Quality of evidence for meta-analyses began at the high level and was downgraded to lower levels of evidence when risk of bias, inconsistency, indirectness, imprecision or publication bias were present. Publication bias was examined by Egger test [27].

### Statistical analysis

Means and standard deviations of change scores for both intervention and control groups were included in one of the extracted studies [28]. Using these change data, the correlation coefficients were calculated for the intervention group (*r* = 0.81) and control group (*r* = 0.80), with an average r of 0.80 [28]. For all included studies, the standard deviation of change scores from baseline were calculated using a correlation coefficient of 0.8 [25], and entered directly into Review Manager 5.3 (Version 5.3; The Cochrane Collaboration, Denmark) for analysis. Analyses based on changes from baseline are more efficient and powerful than comparison of final values through the removal of between-person variability [25]. The mean differences with 95% confidence intervals (CIs) were calculated for anthropometric outcomes. For PA outcomes, standardised mean differences (SMD) with 95% CIs were calculated using Review Manager 5.3 as the mean difference divided by the pooled standard deviation [25]. Due to the heterogeneity in the study interventions and populations, meta-analyses were conducted using a random effects model [25]. In keeping with recommendations, an effect size of 0.2 was considered small, 0.5 moderate, and 0.8 or more was considered large [29]. The effect of heterogeneity of each summary effect size was quantified using a chi-squared test and the I^2^ statistic, in which the boundary limits 25, 50, and 75% were designated as a low, moderate, and high heterogeneity value, respectively [25].

### Sensitivity and subgroup analyses

All analyses were repeated with correlations set at lower (0.50) and higher (1.0) r values than the calculated value of 0.80. Sensitivity analyses were performed to assess heterogeneity of the studies and to evaluate the robustness of the results. Each study was individually removed to evaluate the effect of that study on the summary estimates.

Subgroup analyses were performed to investigate the essential elements in designing effective behaviour change interventions in the ambulatory hospital setting. The subgroup analyses included the study population, follow-up duration, objective or self-reported measurements, the duration of intervention, and the dose of the intervention. The duration of intervention was classified as short term (≤ 3 months) or longer term if ≥4 months [30]. The reporting of the length of intervention sessions was poor in many of the included studies. As a result the intervention dose quantified in this review is through the number of sessions. This intervention dose was categorised as low intensity (≤ 6 sessions), medium intensity (7–12 sessions) or high intensity (≥ 13 sessions) [31].

## Results

**Fig. 1:**
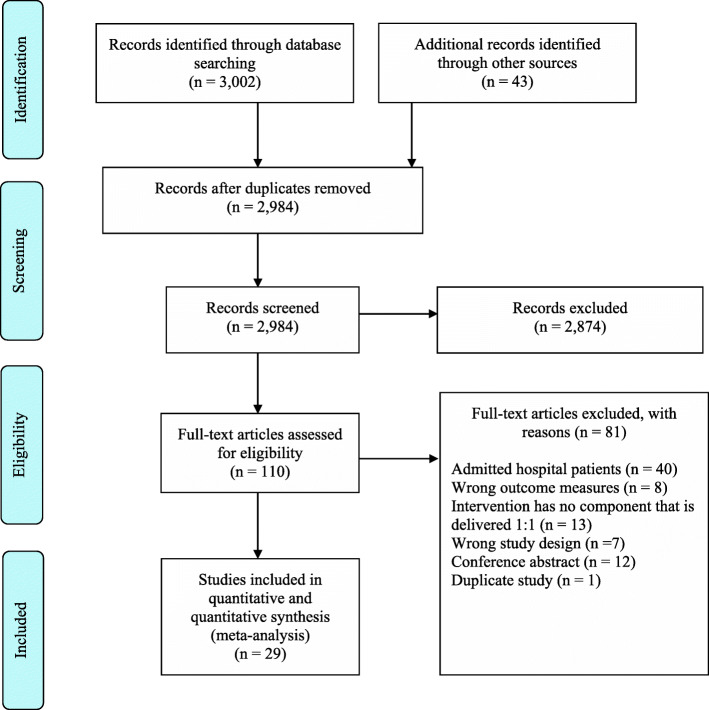
PRISMA

Following de-duplication, 2984 studies were screened. The PRISMA diagram for the screening is shown in Fig. 1. Twenty-nine full-text articles fulfilled the inclusion criteria and were included in qualitative (*n* = 29) and quantitative (*n* = 21) syntheses (Table 1) [28, 32–59]. Included studies were published over a 17-year period from 2003 to 2020. The studies were performed in 14 different countries, with the largest representation from the United States (*n* = 6), Holland (*n* = 4) and Australia (*n* = 4). Populations within the included studies represented various health conditions including impaired glucose tolerance (IGT) or type 2 diabetes (T2DM) (*n* = 10) [32, 36, 37, 40, 48, 49, 53, 55, 56, 59], cardiovascular diseases (CVD) (*n* = 10) [28, 35, 38, 39, 41, 45–47, 50, 51], overweight/obesity (*n* = 5) [42, 44, 52, 54, 57], insufficiently physically active (*n* = 1) [34], Chronic Obstructive Pulmonary Disease (COPD) (*n* = 1) [58], cerebrovascular disease (*n* = 1) [33], and cancer (*n* = 1) [43].

**Table 1:** Characteristics of the included studies

Study	Country	Study Population	N (% male)	Mean age (SD)	Intervention delivery	Underlying Theory ^**a**^	Behaviour change techniques ^**a**^	Length of intervention	Length of follow-up	Outcome measures
Aas, 2005 [32]	Norway	Overweight patients with T2DM	38 (63%)	57 ± 6	14 x group education sessions; 2 x counselling sessions over 12 months, delivered in person; Group exercise sessions delivered twice a week	Not stated	Goal setting	12 months	12 months	Anthropometric: Objective measurement
Ahmadi, 2020 [33]	Germany & Denmark	Patients with cerebrovascular disease	2098 (33%)	67 ± 10	8 x individual counselling sessions over 24 months, delivered in person	MI	Feedback on behaviour; Feedback on outcome	2 years	3 years	Anthropometric: NS; Physical Activity: Self-reported
Alsaleh, 2016 [28]	Jordan	Patients with CVD	156 (53%)	58 ± 9	6 × 15–20 min counselling sessions over 6 months delivered via telephone; Educational text messages were provided 2 per week for first 3 months, and 1 per week for last 3 months	Social Cognitive Theory; Self-Efficacy Theory	Feedback; Goal setting; Self-monitoring; MI techniques	6 months	9 months	Anthropometric: NS; Physical Activity: Self-reported using IPAQ
Altenburg, 2014 [58]	Netherlands	Patients with COPD	155 (65%)	62 ± 4	5 × 30 min counselling sessions over 12 weeks delivered in person	Goal setting and task performance	Goal setting; MI techniques	3 months	15 months	Physical Activity: Pedometer
Barrett, 2018 [34]	Australia	Insufficiently physically active adults	72 (25%)	53 ± 8	1 x group education session; 8 × 30-min individual sessions over 12 weeks, delivered via telephone	Integrated MI and CBT	Goal setting, action planning, self-monitoring, personal feedback; relapse prevention	3 months	6 months	Anthropometric: Objective measurement; Physical Activity: Actigraph Accelerometer
Cakir, 2006 [35]	Turkey	Patients with hypertension	70 (58%)	52 ± 8	1 × 30-min group lecture; 4 × 60-min group education classes; 4 x individual counselling sessions, delivered in person	Not stated	Education; Stress management; Coping strategies	3 months	6 months	Anthropometric: NS; Physical Activity: Self-reported using Health Promoting Lifestyle Profile.
Carrasquillo, 2017 [36]	USA	Latinos with T2DM	300 (45%)	55 ± 7	4 x individual counselling sessions over 12 months, delivered in person; 12 x individual counselling sessions over 12 months, delivered via telephone; Intervention participants were invited to monthly educational groups and bimonthly exercise groups in parks located within a convenient proximity to their homes.	Not stated	MI skills; Education	12 months	12 months	Anthropometric: NS; Physical Activity: Self-reported using IPAQ
Cheung, 2019 [37]	Australia	Post-partum women with GD	60 (0%)	34 ± 4	2 × 30 min individual counselling sessions over 6 months, delivered in person; 1 x follow-up session, up to 12-weeks post-partum, delivered via phone	Focused on the adoption phase of behaviour change	Not stated	6 months	6 months	Anthropometric: Self-reported; Physical Activity: Fitbit
Dogru, 2019 [59]	Turkey	Patients with T2DM	60 (32%)	NS	4 × 15-20 m individual counselling sessions, delivered once a month for 4-months via telephone	MI	MI techniques	4 months	4 months	Anthropometric: Self-reported
Duscha, 2018 [38]	USA	Patients with CVD	25 (76%)	64 ± 8	24 × 30–60 min telephone coaching sessions over 12 weeks delivered in person; In addition, coaches sent educational material via email and sent text messages to remind them to practice healthy lifestyle habits.	Health Coaching	Planning; Motivation	3 months	3 months	Physical Activity: Fitbit
Elkoustaf, 2019 [39]	USA	Patients with CVD	79 (57%)	66 ± 9	1 x groups introduction session; 18 x group sessions over 6 months; 1:1 individual coaching sessions, delivered via phone (unspecified number)	Wellness coaching	Not stated	9 months	9 months	Anthropometric: Objective measurement; Physical Activity: Objective functional measurement
Fappa, 2012 [40]	Greece	Patients with Metabolic Syndrome	87 (42%)	49 ± 12	7 × 60-min counselling sessions over 6 months, delivered in person	Goal setting theory	Self-monitoring; Problem-solving techniques; Relapse prevention	6 months	6 months	Anthropometric: Objective measurement; Physical Activity: Self-reported using Harokopio PA Questionnaire
Freedland, 2015 [41]	USA	Patients with CVD	158 (54%)	56 ± 11	1 × 60-min counselling sessions weekly for the first 6 months, delivered in person; 4 × 30-min counselling sessions in the final 6 months, delivered via phone	CBT	Problem-solving; Goal setting	12 months	12 months	Physical Activity: Objective functional measures
Gade, 2014 [42]	Norway	Patients who were morbidly obese	102 (68%)	43 ± 10	4 x individual counselling session over 10 weeks delivered in person; 6 x individual counselling session over 10 weeks delivered via telephone	CBT	Psychoeducation; Homework; Self-monitoring; Relapse prevention	10 weeks	3 months	Anthropometric: NS
Goedendorp, 2010 [43]	Netherlands	Patients with cancer undergoing curative treatment	240 (34%)	57 ± 11	10 × 60-min counselling sessions over 6 months, delivered in person	CBT	Restructuring of cognitions and beliefs; education; Behavioural instructions	6 months	6 months	Physical Activity: Actometer
Goodwin, 2014 [44]	Canada & USA	Overweight postmenopausal women	338 (0%)	61 ± 7	19 × 30-60 m coaching sessions over 2 years delivered via telephone	Not stated	Lifestyle coaching; Motivation; Relapse prevention; Overcoming barriers	24 months	24 months	Anthropometric: Objective measurement; Physical Activity: Self-reported using IPAQ
Harting, 2006 [45]	Netherlands	Patients with CVD risk	1270 (69%)	61 ± 9	6 × 30–45 min counselling sessions over 4 months, delivered in person	Health Counselling based on stage of behavioural change	Not stated	4 months	18 months	Anthropometric: Objective measurement; Physical Activity: Self-reported using a ‘short validated survey’
Ijzelenberg, 2012 [46]	Netherlands	Patients with CVD	146 (77%)	60 ± 11	22 x group exercise sessions over 6 months; 3 x individual exercise sessions over 6 months; 7 x group counselling sessions over 6 months; Individually counselling sessions over 6 months, delivered in person (unspecified number)	Lifestyle counselling	Motivation; Goal setting; Stress management	6 months	6 months	Anthropometric: Objective measurement; Physical Activity: Self-reported using the SQUASH survey
Kim, 2019 [47]	Korea	Women at risk of CVD	58 (0%)	57 ± 6	12 x individual counselling session over 3 months, delivered in person; 1 x education text message delivered weekly for 3 months	Theory of planned behaviour; Theory of self-regulation	Education; goal setting, self-monitoring; feedback	3 months	3 months	Anthropometric: Objective measurement; Physical Activity: Self-reported using the IPAQ
Kirk, 2004 [48]	UK	Inactive patients with T2DM	70 (50%)	58 ± 8	2 × 30-min individual counselling sessions over 9 months delivered in person; 4 x individual counselling sessions over 9 months delivered via telephone	Transtheoretical model	Problem solving; Social support; Goal setting	9 months	12 months	Physical Activity: Self-reported
Kosaka, 2005 [49]	Japan	Men with IGT	458 (100%)	NS	6 x individual counselling sessions over 12 months, delivered in person	Not stated	Education; Self-monitoring; Social support	12 months	48 months	Anthropometric: Objective measurement.
Lear, 2003 [50]	Canada	Patients with CVD	302 (82%)	64 ± 9	6 x group exercise sessions over 12 months; 2 x lifestyle and risk-factor assessments; 6 x individual counselling sessions over 12 months, delivered in person	Counselling based on principles of behavioural change	Feedback (outcomes); Counsel on lifestyle behaviours and risk factors	12 months	12 months	Anthropometric: Objective measurement; Physical Activity: Self-reported using MLTPA questionnaire.
Miura, 2004 [51]	Japan	Patients with HTN	57 (51%)	62 ± 10	6 x individual counselling sessions over 6 months, delivered in person	Behaviour theory; Social cognitive theory	Not stated	6 months	6 months	Anthropometric: Objective measurement; Physical Activity: Actigraph Accelerometer
O’Brien, 2018 [52]	Australia	Overweight patients with OA	120 (36%)	62 ± 12	1 x brief group education session; 10 x individual counselling session over 6-months, delivered in person	MI; Self-regulation principles	Problem solving; Goal setting	6 months	6 months	Anthropometric: Objective measurement; Physical Activity: Self-reported using AAS
Oldroyd, 2006 [53]	UK	Patients with IGT	78 (50%)	58 ± 10	12 × 15–20 min individual counselling sessions over 24 months, delivered in person	Stages of change model of behaviour change	MI techniques; Action planning; Goal setting	24 months	24 months	Anthropometric: Objective measurement; Physical Activity: Self-reported using a ‘lifestyle questionnaire’
Rimmer, 2009 [54]	USA	Women with morbid obesity & mobility issues	92 (0%)	59 ± 11	1 x individual counselling sessions each week over 6 months, delivered in person; Option to attend a monthly exercise support group.	Not stated	Goal Setting; Performance feedback; Overcoming barriers	6 months	6 months	Anthropometric: Objective measurement; Physical Activity: Self-reported using a ‘lifestyle questionnaire’
Sone, 2010 [55]	Japan	Patients with T2DM	2033 (47%)	59 ± 7	1 x group education session; 2 × 15-min individual counselling session monthly over 96 months, delivered in person	Not stated	Feedback on behaviour; Feedback on outcomes	96 months	96 months	Anthropometric: Objective measurement; Physical Activity: Self-reported using a ‘lifestyle questionnaire’
Wattanakorn, 2013 [56]	Thailand	Patients with T2DM and obesity	76 (16%)	50 ± 8	4 × 30–45 min individual counselling sessions over 1 month, delivered in person	MI; Self-regulation theory.	Education; Goal setting; Discrepancy between current behaviour and goal	1 month	4 months	Anthropometric: Objective measurement; Physical Activity: Self-reported using the Seven Day PA Recall survey
Williams, 2018 [57]	Australia	Overweight patients with chronic LBP	159 (41%)	57 ± 13	10 x individual counselling sessions over 6 months, delivered via telephone	SDT;	Setting graded tasks; Setting specific behaviour goals; Barrier identification Prompting self-monitoring of behaviour and outcomes	6 months	6 months	Anthropometric: Objective measurement; Physical Activity: Self-reported using the AAS

*AAS* Active Australia Survey, *CBT* Cognitive Behaviour Therapy, *COPD* Chronic Obstructive Pulmonary Disease, *CVD* Cardiovascular disease, *HTN* Hypertension, *IGT* Impaired Glucose Tolerance, *IPAQ* International Physical Activity Questionnaire, *LMTPA* Minnesota Leisure Time Physical Activity, *MI* Motivational Interviewing, *NS* Not stated, *OA* Osteoarthritis, *PA* Physical Activity, *SDT* Self-determination Theory, *SQUASH* Short QUestionnaire to ASsess Health enhancing physical activity, *SR* Self-reported, *T2DM* Type 2 Diabetes Mellitus. ^**a**^as described by the authors of the studies

### Study characteristics

The behaviour change interventions in the included studies varied in intervention duration from 4 to 416 weeks. With the exclusion of Sone et al. [55], which used a low grade intervention over 8 years, the adjusted intervention duration was 32 ± 24 weeks. The intervention duration was 26 weeks or greater in 66% of the included studies. Follow-up duration varied amongst the studies: 5 studies had a 3-month follow-up [38, 42, 47, 48, 56], 1 study had a 4-month follow-up [59], 10 studies had a 6-month follow-up [34, 35, 37, 40, 43, 46, 51, 52, 54, 57], 2 studies had a 9-month follow-up [28, 39], 4 studies had a 12-month follow-up, and the remaining 7 studies varied between 15 months and 8 years of follow-up [33, 44, 45, 49, 53, 55, 58].

The intervention components used in the included studies varied. All of the included studies had at least one component that was delivered 1:1. The underlying theory informing the behaviour change intervention and the behaviour change techniques used are detailed in Table 1. For nine studies, the main focus of the intervention was on increasing PA [28, 34, 38, 43, 47, 48, 51, 54, 58]. Changes in anthropometrics was the primary focus in four studies [40, 44, 52, 54].

For PA outcomes, objective measurement was used in 8 studies using accelerometers, pedometers and objective measurement of exercise capacity [34, 37–39, 41, 43, 51, 58]. Self-reported instruments were used in the other 18 studies [28, 33, 35, 36, 40, 41, 44–48, 50, 52–57]. The measures of anthropometrics in the studies included body mass [28, 32, 34, 35, 44, 52–54, 57], BMI [28, 32–36, 39, 40, 50, 52–55, 57, 59] and WC [34, 35, 40, 50, 53]. Objective measurement of anthropometrics was used in 15 of the studies [32, 34, 39, 44, 46, 47, 49–57], with self-reported methods used in the remaining 3 studies [37, 45, 59].

The professional background of the persons delivering the interventions included community health workers [36], dietitians [37, 38, 40, 50, 53, 55], exercise counsellors [51, 58], exercise scientists [48, 50], graduate level therapists [41], health professionals [32], health educators [38, 39], lifestyle coaches [44], nurses [28, 33, 35, 45, 55], physicians [33, 55], physiotherapists [34, 53, 55], psychologists [46], researchers [56, 57, 59], and therapists [43].

### Risk of bias

**Fig. 2:**
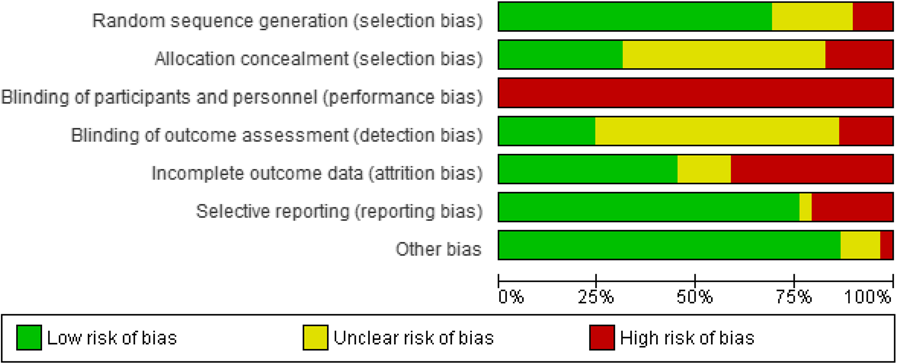
Risk of bias of included studies

The risk of bias assessment for all studies is detailed in Fig. 2. In trials involving behaviour change interventions the blinding of participants is extremely difficult to undertake. As a result, all studies were judged to have a high of risk of performance bias (lack of blinding of participants and personnel). Twelve studies were judged to have a high risk of attrition bias, and four studies were rated as unclear. Seven of the included studies reported blinding of the outcome assessors (detection bias), whereas the majority of the studies did not adequately report blinding of the outcome assessors (*n* = 18). Five of the included studies were judged as a high risk of selection bias due to the lack of detail regarding the allocation concealment. Fifteen studies were judged to have an unclear risk of bias due to the lack of information provided on the random sequence generation. The individual risk of bias assessment is included in Additional file 3.

### GRADE assessment

The overall certainty of evidence for the effectiveness of behaviour change interventions for changes in PA and anthropometrics in adults attending ambulatory hospital clinics is presented in Table 2. The certainty of evidence for the meta-analysis stratified by follow-up duration and for studies with a low risk of bias are presented in Additional file 4. In addition, the GRADE quality assessments are presented in Additional file 5.

**Table 2:** Summary of findings table

Behaviour change interventions for changes and maintenance in PA and anthropometrics in adults attending ambulatory hospital clinics				
Outcome	Anticipated absolute effects* (95% CI)	№ of participants (studies)	Certainty of the evidence (GRADE)	Informative statements
Physical activity	SMD 0.96 higher [0.45 to 1.48]	1454 (13 RCTs)	⨁◯◯◯ VERY LOW ^a,b,c,d,e^	Behaviour change interventions may increase physical activity in ambulatory hospital patients but the evidence is very uncertain.
Mass (kg)	MD − 2.74 lower [− 4.42 to − 1.07]	872 (9 RCTs)	⨁◯◯◯ VERY LOW ^a,c,d,e,f^	The evidence is very uncertain about the effect of behaviour change interventions on changes in mass in ambulatory hospital patients.
BMI (kg/m^2^)	MD − 0.99 lower [− 1.48 to − 0.50]	4728 (15 RCTs)	⨁◯◯◯ VERY LOW ^a,b,c,d,e^	Behaviour change interventions may decrease BMI in ambulatory hospital patients but the evidence is very uncertain.
Waist Circumference (cm)	MD − 2.21 lower [− 4.01, − 0.42]	530 (5 RCTs)	⨁◯◯◯ VERY LOW ^a,c,d,f^	The evidence is very uncertain about the effect of behaviour change interventions on changes in waist circumference in ambulatory hospital patients.

*The risk in the intervention group (and its 95% confidence interval) is based on the assumed risk in the comparison group and the **relative effect** of the intervention (and its 95% CI)

**Explanations**

^a^Large number of studies with high risk of bias

^b^High heterogeneity

^c^Differences in population and outcome measures

^d^Wide confidence intervals

^e^Asymmetry in the pattern of results

^f^Moderate heterogeneity

### Effects of behaviour change interventions on changes in physical activity

**Fig. 3:**
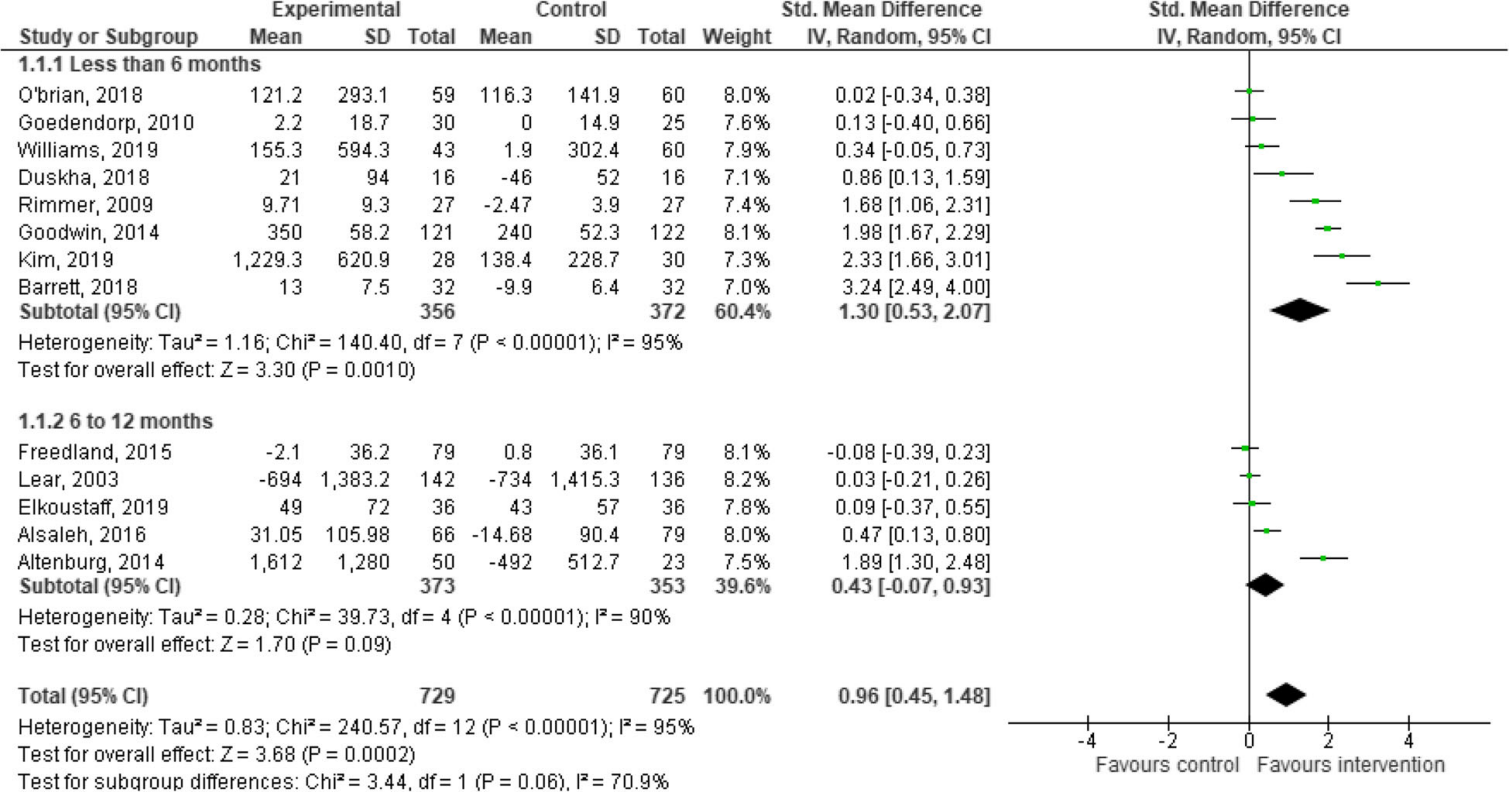
Meta-analysis investigating behavioural lifestyle interventions for changes in physical activity

Thirteen of the 29 included studies provided PA data for the intervention and control groups at the post-intervention follow-up, and were included in the meta-analysis. The meta-analysis for behaviour change interventions versus standard care for change in PA demonstrated a significant effect in favour of the intervention (SMD: 0.96; 95% CI: 0.45 to 1.48, *p* < 0.01, Fig. 3) [28, 34, 38, 39, 41, 43, 44, 47, 50, 52, 54, 57, 58].

Subgroup analyses indicated that behaviour change interventions resulted in a significant increase in PA when the follow-up lasted for 6 months or less (SMD: 1.30; 95% CI: 0.53 to 2.07, *p* < 0.01, Fig. 3) [34, 38, 43, 44, 47, 52, 54, 57]. Behaviour change interventions with a follow-up of greater than 6 months demonstrated a non-significant effect in favour of the intervention (SMD: 0.43; 95% CI: − 0.07 to 0.93, *p* = 0.09, Fig. 3) [28, 39, 41, 50, 58]. Behaviour change interventions may increase PA in ambulatory hospital patients but the evidence is very uncertain.

### Effects of behaviour change interventions on changes in body mass

**Fig. 4:**
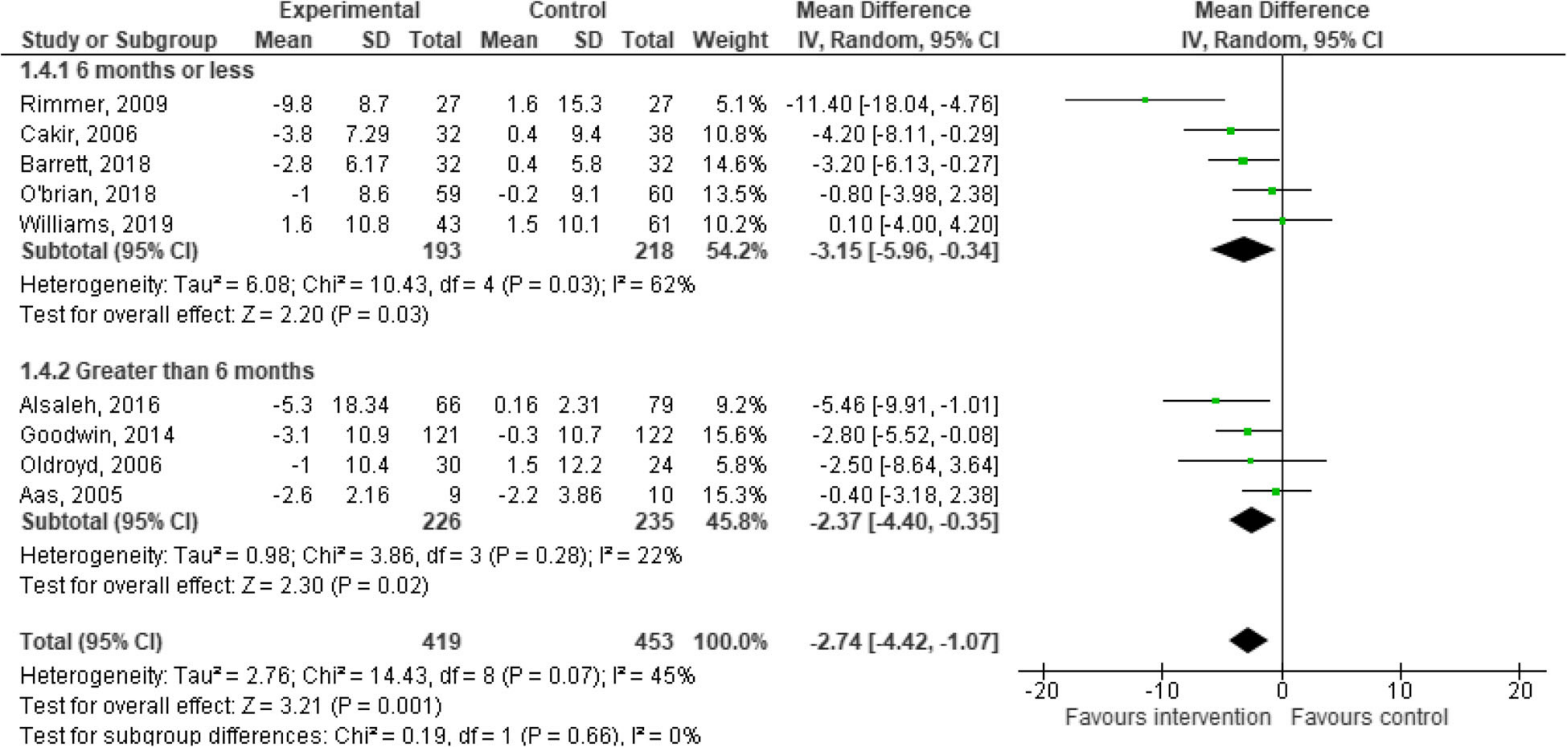
Meta-analysis investigating behavioural lifestyle interventions for changes in body mass

Nine studies provided data on changes in body mass for the experimental and control groups at the post-intervention follow-up, and were included in the meta-analysis. The meta-analysis for behaviour change interventions versus standard care for change in body mass demonstrated a significant effect in favour of the intervention (MD: -2.74; 95% CI: − 4.42 to − 1.07, *p* < 0.01, Fig. 4) [28, 32, 34, 35, 44, 52–54, 57].

Subgroup analyses indicated that behaviour change interventions resulted in a significant changes in body mass when follow-up measurement was 6 months and under (MD: -3.15; 95% CI: − 5.96 to − 0.34, *p* = 0.03, Fig. 4), and greater than 6 months (MD: -2.37; 95% CI: − 4.40 to − 0.35, *p* = 0.02, Fig. 4). The evidence is very uncertain about the effect of behaviour change interventions on changes in mass in ambulatory hospital patients.

### Effects of behaviour change interventions on changes in BMI

**Fig. 5:**
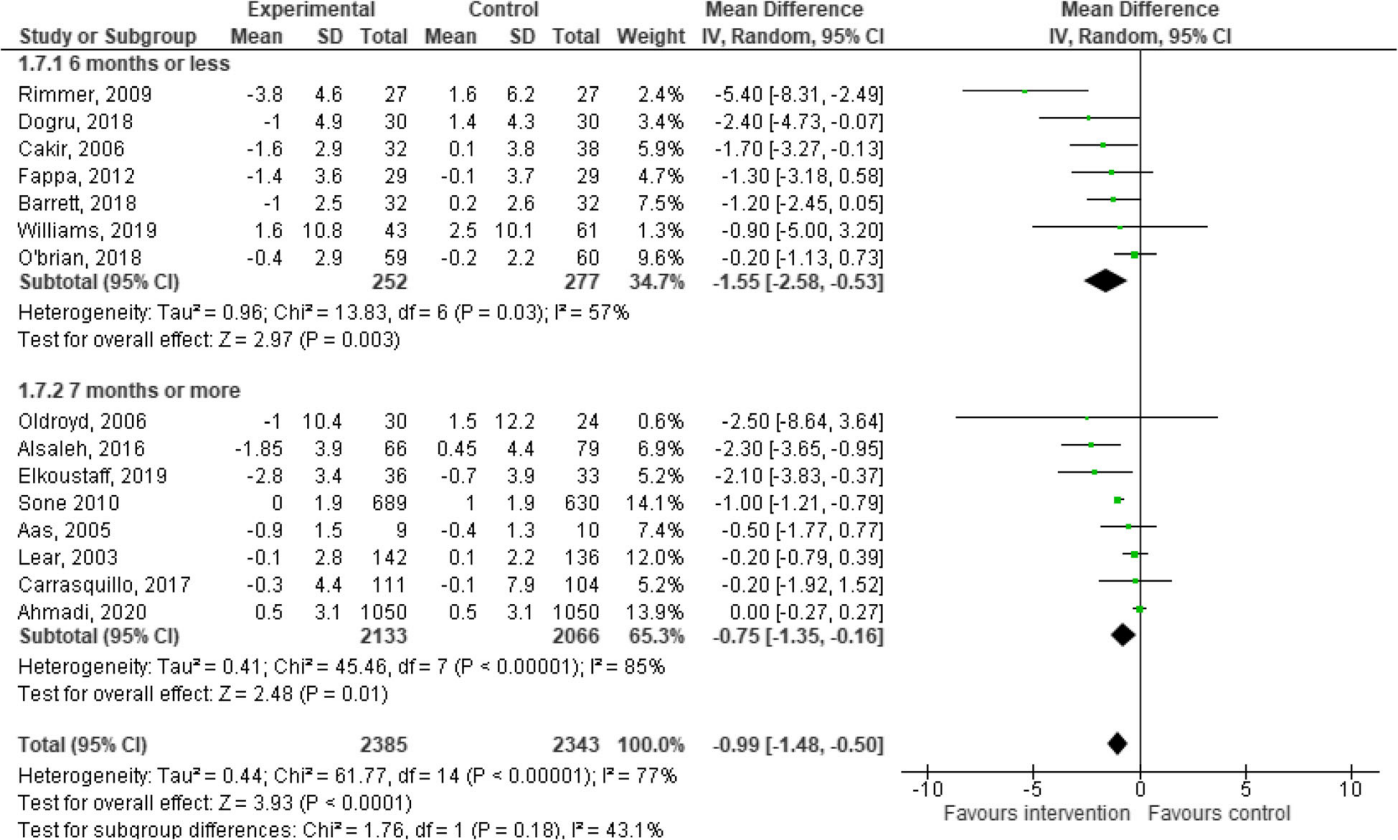
Meta-analysis investigating behavioural lifestyle interventions for changes in BMI

Fifteen studies provided data on changes in BMI for the experimental and control groups at the post-intervention follow-up and were included in the meta-analysis. The meta-analysis for behaviour change interventions versus standard care for change in BMI demonstrated a significant effect in favour of the intervention (MD: -0.99; 95% CI: − 1.48 to − 0.50, *p* < 0.01, Fig. 5) [28, 32–36, 39, 40, 50, 52–55, 57, 59].

The behaviour change interventions demonstrated significant changes in BMI when follow-up measurement was 6 months and under (MD: -1.55; 95% CI: − 2.58 to − 0.53, *p* < 0.01, Fig. 5), and greater than 6 months (MD: -0.75; 95% CI: − 1.35 to − 0.16, *p* = 0.01, Fig. 5). Behaviour change interventions may decrease BMI in ambulatory hospital patients but the evidence is very uncertain.

### Effects of behaviour change interventions on changes in waist circumference

**Fig. 6:**
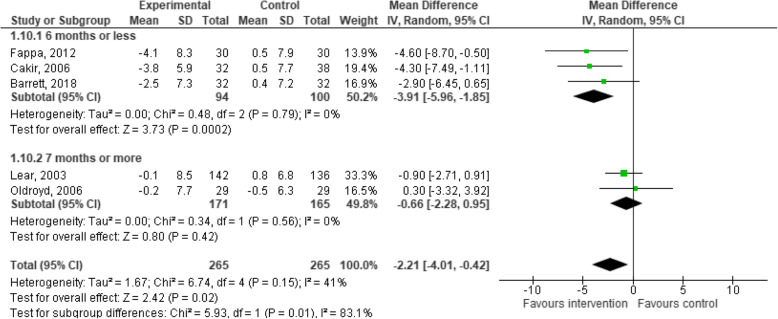
Meta-analysis investigating behavioural lifestyle interventions for changes in waist circumference

Five studies provided data on changes in WC for the experimental and control groups at the post-intervention follow-up and were included in the meta-analysis. The meta-analysis for behaviour change interventions versus standard care for change in WC demonstrated a significant effect in favour of the intervention (MD: -2.21; 95% CI: − 4.01 to − 0.42, *p* = 0.02, Fig. 6) [34, 35, 40, 50, 53].

The behaviour change interventions demonstrated significant changes in WC when follow-up measurement was 6 months and under (3 studies, 194 participants, MD, − 3.91, 95% CI, − 5.96 to − 1.85, *p* < 0.01, Fig. 6), but not when the follow-up was greater than 6 months (MD: -0.66; 95% CI: − 2.88 to 0.95, *p* = 0.42, Fig. 6). The evidence is very uncertain about the effect of behaviour change interventions on changes in WC in ambulatory hospital patients. The one exception was the analysis for WC change when follow-up measurement was 6 months and under, in which case the evidence suggests that behaviour change interventions results in a slight reduction in WC in ambulatory hospital patients.

### Sensitivity and subgroup analyses

Sensitivity analyses of the imputed correlation coefficients revealed that effect sizes remained statistically significant, and within the 95% confidence intervals at the imputed r of 0.8 (Table 3). Statistically significant changes remained for all outcomes at the inputed r of 1.0 and 0.5. In the low risk of bias analyses, behaviour change interventions exhibited significant beneficial effects in PA, body mass and BMI. Subgroup analyses demonstrated significant changes in body mass and BMI for individuals with cardiovascular diseases, and significant changes in BMI for individuals with type 2 diabetes/impaired glucose tolerance (Table 3). Larger effect sizes were observed for PA and BMI changes when objective measurement was used. Larger effect sizes were observed for changes in body mass when self-reported measurement was used. No subgroup analysis could be conducted on changes in WC between objective and self-reported measures.

**Table 3:** Sensitivity and subgroup analyses

Characteristics	No. of studies	No. of participants (intervention/control)	Mean change (95% confidence interval)	***p***-value	Heterogeneity
**Physical activity**					
Full analysis	13	1454 (729/725)	0.96 [0.45, 1.48]	< 0.01	95%
Excluding high risk of bias overall	5	677 (340/337)	1.04 [0.15, 1.92]	0.02	96%
Objective measurement	4	224 (128/96)	1.52 [0.22, 2.81]	0.02	94%
Self-reported measurement	9	1230 (601/629)	0.74 [0.17, 1.30]	0.01	95%
*r* = 0.5	13	1454 (729/725)	0.72 [0.32, 1.13]	< 0.01	92%
*r* = 1.0	13	1454 (729/725)	1.86 [0.96, 2.76]	< 0.01	98%
Short-term intervention duration	4	235 (126/101)	2.08 [1.18, 2.97]	< 0.01	86%
Long-term intervention duration	9	1227 (603/624)	0.51 [0.00, 1.02]	0.05	94%
Low intensity intervention	4	654 (337/317)	0.52 [−0.08, 1.12]	0.09	92%
Medium intensity intervention	6	642 (313/329)	1.31 [0.35, 2.28]	< 0.01	96%
High intensity intervention	3	158 (79/79)	0.86 [−0.13, 1.85]	0.09	88%
Obese subgroup	4	619 (250/269)	1.00 [0.04, 2.04]	0.06	96%
CVD subgroup	5	675 (339/346)	0.19 [−0.07, 0.45]	0.15	61%
**Body Mass (kg)**					
Full analysis	9	872 (419/453)	-2.74 [−4.42, −1.07]	< 0.01	45%
Excluding high risk of bias overall	3	253 (123/130)	−2.59 [− 4.49, −0.68]	< 0.01	2%
Objective measurement	7	656 (321/336)	− 2.25 [−4.16, −0.34]	0.02	48%
Self-reported measurement	2	226 (98/117)	−4.75 [−7.69, −1.81]	< 0.01	0%
*r* = 0.5	9	872 (419/453)	−2.43 [−4.18, −0.69]	< 0.01	0%
*r* = 1.0	9	872 (419/453)	−2.21 [−3.57, −0.84]	< 0.01	98%
Short-term intervention duration	2	134 (64/70)	−3.56 [−5.91, −1.21]	< 0.01	0%
Long-term intervention duration	7	738 (355/383)	−2.57 [−4.57, −0.38]	0.04	54%
Low intensity intervention	1	135 (66/79)	−5.46 [−9.91, −1.01]	0.02	NA
Medium intensity intervention	5	411 (196/215)	−2.14 [−3.80, −0.49]	0.01	0%
High intensity intervention	3	316 (157/159)	−3.82 [−8.26, 0.63]	0.09	78%
Obese subgroup	4	520 (250/270)	−2.85 [−6.27, 0.56]	0.10	68%
CVD subgroup	2	215 (98/117)	−4.75 [−7.69, −1.81]	< 0.01	0%
Diabetes/IGT subgroup	2	73 (39/34)	−0.76 [−3.29, 1.77]	0.56	0%
**BMI (kg/m**^**2**^**)**					
Full analysis	15	4728 (2385/2343)	−0.99 [−1.48, − 0.50]	< 0.01	77%
Excluding high risk of bias overall	4	531 (265/266)	−0.57 [−1.20, 0.05]	0.07	37%
Objective measurement	11	2198 (1126/1072)	−0.99 [−1.52, − 0.47]	< 0.01	55%
Self-reported measurement	4	2530 (1259/1271)	−0.97 [−2.24, 0.29]	0.13	80%
*r* = 0.5	15	4728 (2385/2343)	−0.88 [−1.43, − 0.34]	< 0.01	59%
*r* = 1.0	15	4728 (2385/2343)	−1.38 [− 1.88, − 0.88]	< 0.01	98%
Short-term intervention duration	2	134 (64/70)	−1.39 [−2.37, − 0.42]	< 0.01	0%
Long-term intervention duration	13	4594 (2321/2273)	−0.93 [−1.47, − 0.39]	< 0.01	80%
Low intensity intervention	4	698 (349/349)	−1.12 [−2.38, 0.15]	0.02	71%
Medium intensity intervention	7	2569 (1275/1269)	−0.56 [−1.17, 0.04]	0.07	39%
High intensity intervention	4	1461 (761/700)	−1.63 [−2.85, −0.42]	< 0.01	72%
Obese subgroup	3	277 (129/148)	−2.08 [−5.56, 1.40]	0.24	82%
CVD subgroup	2	139 (68/71)	−1.88 [−3.04, −0.72]	< 0.01	0%
Diabetes/IGT subgroup	6	1725 (898/827)	−0.99 [−1.19, − 0.79]	< 0.01	0%
**Waist Circumference (cm)**					
Full analysis	5	530 (265/265)	−2.21 [−4.01, −0.42]	0.02	41%
Excluding high risk of bias overall	4	472 (236/236)	−2.34 [− 4.49, −0.18]	0.03	45%
Objective measurement	4	460 (233/227)	−1.64 [−3.43, 0.15]	0.07	28%
Self-reported measurement	–	–	–	–	–
*r* = 0.5	5	530 (265/265)	−2.40 [−4.20, −0.59]	0.02	55%
*r* = 1.0	5	530 (265/265)	−2.61 [−4.23, −0.99]	< 0.01	99%
Short-term intervention duration	2	134 (64/70)	−3.68 [−6.05, −1.30]	< 0.01	0%
Long-term intervention duration	3	396 (201/195)	−1.40 [−3.68, 0.88]	0.23	41%
Low intensity intervention	1	278 (142/136)	−0.90 [−2.71, 0.91]	0.33	NA
Medium intensity intervention	4	252 (123/129)	−2.87 [−5.04, −0.70]	0.01	32%
High intensity intervention	–	–	–	–	–
CVD subgroup	2	348 (174/174)	−2.34 [−5.63, 0.96]	0.40	70%
Diabetes/IGT subgroup	2	118 (59/59)	−2.05 [−6.85, 2.75]	0.06	53%

*BMI* Body Mass Index, *CVD* Cardiovascular Disease, *IGT* Impaired Glucose Tolerance, *NA* Not applicable

Interventions with a short-term duration demonstrated significant effects for changes in PA, body mass, BMI and WC (Table 3). Interventions with a longer-term duration demonstrated significant effects for changes in body mass and BMI only (Table 3). In terms of intervention dose, interventions categorised as low intensity demonstrated significant effects for changes in body mass and WC (Table 3). Interventions categorised as medium intensity demonstrated significant effects for changes in PA and body mass (Table 3). Interventions categorised as high intensity demonstrated significant effects for changes in BMI (Table 3). No subgroup analysis could be conducted on changes in WC between dose of intervention. The moderate to high heterogeneity found in the primary meta-analyses was consistent across the majority of sensitivity and subgroup analyses.

## Discussion

This systematic review and meta-analyses provides evidence to support the use of behaviour change interventions for changes in PA and anthropometrics, initiated in the ambulatory hospital setting. The effect sizes were large for PA and moderate for anthropometric outcomes. These positive results are important as even small positive changes in PA and anthropometrics can deliver beneficial health benefits [60]. The moderate to large effect sizes demonstrated here are likely to deliver important health outcomes for ambulatory hospital patients [60]. Patients attending secondary care hospital clinics are more likely than the general population to have preventable chronic disease due to risk factors such as insufficient PA or overweight and obesity [61]. Behaviour change interventions aimed at changes in PA and anthropometrics can go towards addressing health risks in this population [62]. Nevertheless, the heterogeneity of results for all outcomes were moderate to high, and the GRADE assessment indicated that the evidence is very uncertain about the effect of behaviour change interventions on changes in PA and anthropometrics.

The meta-analysis of 13 randomised controlled trials for behaviour change interventions versus standard care for changes in PA demonstrated a significant large effect (*d* = 0.96) in favour of the intervention. The effect size is larger than those reported for PA interventions aiming to increase PA in older adults (*d* = 0.26) [63], chronically ill adults (*d* = 0.45) [64], healthy inactive adults (*d* = 0.32) [65] and young and middle aged adults (*d* = 0.32) [66], but similar to that reported for behaviour change interventions targeting individuals at risk of cardiovascular disease [10, 11]. The heterogeneity of both interventions and outcome measures, and the wide confidence intervals observed in the included studies contributed to the downgrading of the certainty about the results to very low. Despite the low level of certainty, it is encouraging to see a significant positive intervention effect across the diverse clinical populations with the included measures of PA participation.

When stratified by follow-up duration, the analyses of the effect of behaviour change interventions on changes in PA demonstrated a significant increase in PA when the follow-up lasted for 6 months sessions or less. Interventions with a follow-up of greater than 6 months demonstrated a non-significant effect in favour of the intervention. Samdal et al., (2017) found that strategies such as motivational interviewing and goal setting are effective for assisting individuals in initiating PA behaviour change [67]. Cognitive strategies such as problem solving and relapse prevention, on the other hand, promote changes in cognition, PA beliefs and influence behaviour change maintenance [68]. Some of the most common strategies used in the studies included in this review were motivational interviewing, goal setting and general counselling/health coaching. These strategies are all acknowledged as important theoretical constructs for successful behaviour change [69]; however, very few of the included studies clearly demarcated the use of strategies for PA maintenance, which could have impacted the effect size over the longer term follow-up. Only a small number of the included studies aimed to engage participants in existing community resources. Referrals to specific community programs, such as walking groups, strength training, and exercise for adults, have shown to have a positive effect on longer-term PA behaviour [69].

The meta-analyses of behaviour change interventions versus standard care for changes in anthropometric outcomes demonstrated significant positive effects in body mass, BMI and WC. Significant reductions in body mass, BMI and WC were found when the follow-up lasted for 6 months or less. Significant favourable changes in body mass and BMI were found when the follow-up lasted for greater than 6 months. The increasing prevalence of overweight and obesity over recent decades have been a major public health concern [70]. Overweight and obesity not only have a direct impact on morbidity, but contribute significantly towards further metabolic conditions, including insulin resistance, and type 2 diabetes [71]. Behaviour change interventions, predominantly focusing on changes in PA and anthropometrics, are the central tenets of prevention programs needed to address overweight and obesity prevalence [72]. This review adds to the evidence base to support the use of behaviour change interventions to influence anthropometric changes in the ambulatory hospital setting.

The 2.74 kg (95% CI: − 4.42 to − 1.07) reduction in body mass found in this meta-analysis compares to similar reductions of 3.77 kg (95% CI: − 4.55 to − 2.99) [73] and 2.12 kg (95% CI: − 2.61 to − 1.63) [74] found in behaviour change interventions for people at high risk for diabetes, and in nutritional education programs with a specific focus on weight loss (− 2.07 kg; 95% CI: − 1.52 to − 2.62). The mean reduction in BMI of 0.99 kg/m^2^ (95% CI: − 1.48 to − 0.50) found in this meta-analysis lies between the results from studies in secondary prevention behaviour change interventions, being − 0.16 kg/m^2^ (95% CI: − 0.62 to 0.31) [10] and − 1.80 kg/m^2^ (95% CI: − 2.62 to − 0.99) [11]. The significant decrease in body mass and BMI over the longer-term follow-up is noteworthy given the mean age of the individuals in the analyses was 57. High proportions of middle aged individuals continue to gain weight each year [75]. The magnitude of improvements observed for changes in anthropometrics found in this review are likely to be clinically significant. Favourable changes in anthropometrics are associated with decreased risk for cardiovascular events [76], type 2 diabetes [76, 77] and some cancers [77].

### Implications for practice

Previous research has shown that experiencing health events such as hospital appointments can be the catalyst for changes in behaviour [15, 78]. Ambulatory hospital patients represent an ideal population to intervene with to lessen the risk of developing serious health conditions. Incorporating the use of behaviour change interventions to increase PA in adults attending ambulatory hospital clinics aligns with the 2020 World Health Organization guidelines on PA and sedentary behaviour, which indicate the importance of PA for individuals with chronic conditions [79]. The current analysis incorporates a wide range of participant populations attending ambulatory hospital clinics, ranging from younger to older adults, as well as individuals with health risk factors to individuals with diagnosed chronic conditions. Hospital patients have indicated that they would like the healthcare system to provide guidance on behaviour change and healthy lifestyles [80]. Patients and public health at large might benefit from hospitals shifting their focus from predominantly curative care to a position of more holistic health promotion [81, 82].

Hospitals considering integrating behaviour change interventions into routine care may be encouraged that the delivery of short duration interventions results in statistically significant changes in PA, body mass, BMI and WC for hospital patients. The subgroup analyses provide some indication of the effect of intervention dose on PA and anthropometric changes, with significant changes observed for medium and high intensity interventions. Behaviour change interventions providing a higher number of sessions have been demonstrated to increase self-management skills, which may result in the significant outcomes observed for medium and high intensity interventions [83, 84]. Another potential advantage highlighted in this review was the range of health professionals that were able to deliver the behaviour change intervention. The diversity in clinicians might be advantageous when applying the intervention across differing sectors of the ambulatory hospital setting.

### Limitations

This review has a number of limitations. The wide range of PA measures used within the interventions suggest that caution should be applied when interpreting the translatability of these results. Additionally, only 4 of the studies in the PA meta-analysis used objective measurement [34, 38, 43, 58]. Social desirability bias can lead to over-reporting of PA levels in self-reported measures [85]. Although the majority of self-report questionnaires were based on valid and reliable measures, objective measurements have demonstrated a higher degree of reproducibility and validity for quantifying duration and intensity of PA [86]. The effect size calculated from studies that used objectively measured PA was higher than the overall effect size observed for PA change (Table 3), which improves confidence in the effectiveness of behaviour change interventions to increase PA in ambulatory hospital settings.

The meta-analyses included studies with small sample sizes, and differences in the duration of interventions. The review also included studies with heterogeneous intervention components including differences in the frequency and duration of the sessions, and differences in the professionals providing the intervention. The heterogeneity also existed in the delivery format, including face-to-face, telephone calls and group counselling delivery. This heterogeneity makes the independent contribution of any of the intervention components, or a combination of these factors, difficult to establish, and partially explains the moderate to high heterogeneity of the meta-analyses. The moderate to high heterogeneity was reported in the majority of sub-group analyses, indicating a consistency of results across the examination of the different components of the interventions. Behaviour change interventions tend to exhibit both clinical and methodological diversity, often resulting in statistical heterogeneity within the meta-analyses [87]. Indeed, almost one third of meta-analyses have been shown to result in moderate to high heterogeneity [87]. Finally, only 5 of the 29 included studies reported on intervention fidelity [33, 34, 36, 41, 44]. Without a clear measurement of fidelity, reports of the effectiveness of interventions must be interpreted cautiously, as the possibility that the intervention was not delivered as intended cannot be ruled out [65].

## Conclusion

This review indicates that behaviour change interventions resulted in large improvements in PA, and moderate changes in anthropometric outcomes in adults presenting to ambulatory hospital clinics. The results indicate the value of behaviour change interventions for mitigating chronic disease risk factors, and supports the implementation of behaviour change interventions in ambulatory secondary care clinics. The heterogeneity in study populations, reported outcomes, and intervention components downgraded the certainty of the evidence, and prevents the drawing of firmer conclusions from the evidence provided. In order to improve the translation of these findings into clinical practice, future studies of behaviour change interventions should include clearly defined interventions and assessments of treatment fidelity.

## Supplementary Information

**Additional file 1.**

**Additional file 2.**

**Additional file 3.**

**Additional file 4.**

**Additional file 5.**

## Data Availability

The datasets used and/or analysed during the current study are available from the corresponding author on reasonable request.

## Abbreviations

BMI: Body Mass Index
CI: Confidence Interval
CVD: Cardiovascular disease
IGT: Impaired Glucose Tolerance
MD: Mean Difference
PA: Physical Activity
PRISMA: Preferred Reporting Items for Systematic Reviews and Meta-Analyses
SMD: Standardised Mean Difference
T2DM: Type 2 Diabetes
WC: Waist Circumference
