# Supplementary material for: The effect of behaviour change interventions on changes in physical activity and anthropometrics in ambulatory hospital settings: a systematic review and meta-analysis
Source: Int J Behav Nutr Phys Act. 2021 Jan 7;18:7. doi: 10.1186/s12966-020-01076-6 (PMC7791684; doi:10.1186/s12966-020-01076-6)
Supplement: Supplementary file 2 — Additional file 2. [file 12966_2020_1076_MOESM2_ESM.docx]

Search MEDLINE

| **#** | **Searches** | **Result** | **Type** |
| --- | --- | --- | --- |
| 1 | Hospitals/ | 77929 | Advanced |
| 2 | Outpatients/ | 15910 | Advanced |
| 3 | Outpatient Clinics, Hospital/ | 15573 | Advanced |
| 4 | Ambulatory Care/ | 42903 | Advanced |
| 5 | 1 or 2 or 3 or 4 | 147509 | Advanced |
| 6 | Health Behavior/ | 49806 | Advanced |
| 7 | health behav* change.mp. | 1762 | Advanced |
| 8 | Exercise/ or Walking/ or Health Promotion/ or Motivation/ | 266728 | Advanced |
| 9 | lifestyle change.mp. | 1713 | Advanced |
| 10 | Preventive Health Services/ | 13424 | Advanced |
| 11 | lifestyle intervention.mp. | 4362 | Advanced |
| 12 | Secondary Prevention/ | 20297 | Advanced |
| 13 | Health Promotion/ or Obesity/ or health coach*.mp. | 249606 | Advanced |
| 14 | Telephone/ or Health Promotion/ or telephone coach*.mp. or Self Care/ or Mentoring/ | 117804 | Advanced |
| 15 | 6 or 7 or 8 or 9 or 10 or 11 or 12 or 13 or 14 | 536284 | Advanced |
| 16 | Exercise/ | 108971 | Advanced |
| 17 | physical activity.mp. or Exercise/ | 182310 | Advanced |
| 18 | (weight or mass).mp. [mp=title, abstract, original title, name of substance word, subject heading word, floating sub-heading word, keyword heading word, organism supplementary concept word, protocol supplementary concept word, rare disease supplementary concept word, unique identifier, synonyms] | 2084460 | Advanced |
| 19 | obesity.mp. or Obesity/ | 323560 | Advanced |
| 20 | Metabolic Syndrome/ or Waist Circumference/ or waist circum*.mp. or Obesity/ or Body Mass Index/ | 295558 | Advanced |
| 21 | body mass index.mp. or Body Mass Index/ | 238801 | Advanced |
| 22 | 16 or 17 or 18 or 19 or 20 or 21 | 2368023 | Advanced |
| 23 | 5 and 15 and 22 | 1200 | Advanced |
| 24 | limit 23 to "all adult (19 plus years)" | 854 | Advanced |
