# Supplementary figures and images for: The effect of behaviour change interventions on changes in physical activity and anthropometrics in ambulatory hospital settings: a systematic review and meta-analysis
Source: Int J Behav Nutr Phys Act. 2021 Jan 7;18:7. doi: 10.1186/s12966-020-01076-6 (PMC7791684; doi:10.1186/s12966-020-01076-6)

Additional file


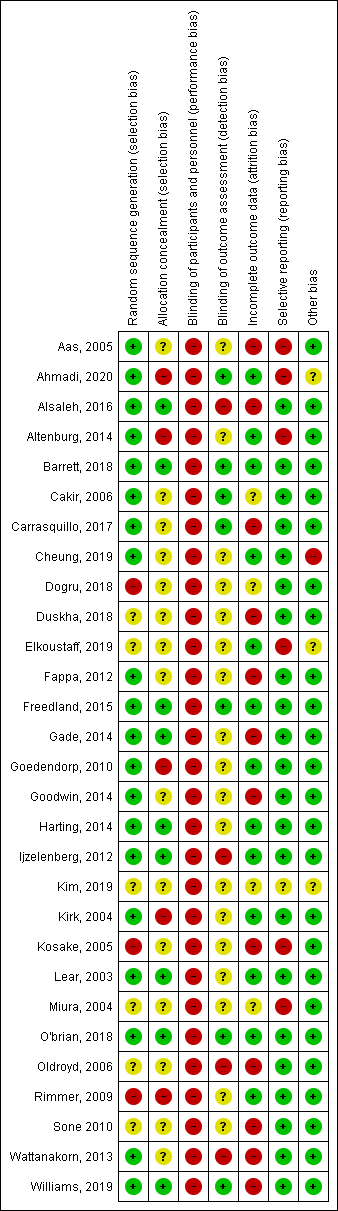


Risk of bias summary for all included studies

Supplement: Supplementary file 3 — Additional file 3. [file 12966_2020_1076_MOESM3_ESM.docx]
