# Supplementary material for: The effect of behaviour change interventions on changes in physical activity and anthropometrics in ambulatory hospital settings: a systematic review and meta-analysis
Source: Int J Behav Nutr Phys Act. 2021 Jan 7;18:7. doi: 10.1186/s12966-020-01076-6 (PMC7791684; doi:10.1186/s12966-020-01076-6)
Supplement: Supplementary file 4 — Additional file 4. [file 12966_2020_1076_MOESM4_ESM.docx]

**Additional file 3** Summary of findings table

| Behaviour change interventions for changes and maintenance in PA and anthropometrics in adults attending ambulatory hospital clinics | | | | |
| --- | --- | --- | --- | --- |
| **Outcome** | **Anticipated absolute effects* (95% CI)** | **№ of participants  (studies)** | **Certainty of the evidence (GRADE)** | **Informative statements** |
| Physical Activity (<=6mo follow-up) | SMD 1.30 higher  [0.53 to 2.07] | 728 (8 RCTs) | ⨁◯◯◯ VERY LOW ^a,b,c,d,e^ | The evidence is very uncertain about the effect of behaviour change interventions on physical activity change in ambulatory hospital patients when the follow-up is 6 months or less. |
| Physical Activity  (>6mo follow-up) | SMD 0.43 higher  [-0.07 to 0.93] | 726 (5 RCTs) | ⨁◯◯◯ VERY LOW ^a,b,c,d,e^ | The evidence is very uncertain about the effect of behaviour change interventions on physical activity change in ambulatory hospital patients when the follow-up is greater than 6 months. |
| Physical activity  (low risk of bias) | SMD 1.04 higher  [0.15, 1.92] | 677 (5 RCTs) | ⨁◯◯◯ VERY LOW ^b,c,e^ | The evidence is very uncertain about the effect of behaviour change interventions on physical activity change in ambulatory hospital patients when studies with a high risk of bias are excluded. |
|  |  |  |  |  |
| Mass (kg)  (<=6mo follow-up) | MD -3.15 lower  [-5.96 to -0.34] | 411 (5 RCTs) | ⨁◯◯◯ VERY LOW ^a,c,d,e,f^ | The evidence is very uncertain about the effect of behaviour change interventions on changes in mass in ambulatory hospital patients when the follow-up is 6 months or less. |
| Mass (kg)  (>6mo follow-up) | MD -2.37 lower  [-4.40 to -0.35] | 461 (4 RCTs) | ⨁◯◯◯ VERY LOW ^a,c,d^ | The evidence is very uncertain about the effect of behaviour change interventions on changes in mass in ambulatory hospital patients when the follow-up is greater than 6 months. |
| Mass (kg)  (low risk of bias) | MD -2.59 lower  [-4.49 to -0.68] | 253 (3 RCTs) | ⨁◯◯◯ VERY LOW ^c,d^ | The evidence is very uncertain about the effect of behaviour change interventions on changes in mass in ambulatory hospital patients when studies with a high risk of bias are excluded. |
|  |  |  |  |  |
| BMI  (<=6mo follow-up) | MD -1.55 lower  [-2.58 to -0.53] | 529 (7 studies) | ⨁◯◯◯ VERY LOW ^a,c,d,e,f^ | The evidence is very uncertain about the effect of behaviour change interventions on changes in BMI in ambulatory hospital patients when the follow-up is 6 months or less. |
| BMI  (>6mo follow-up) | MD -0.75 lower  [-1.35 to -0.16] | 4199 (8 RCTs) | ⨁◯◯◯ VERY LOW ^a,b,c,d,e^ | The evidence is very uncertain about the effect of behaviour change interventions on changes in BMI in ambulatory hospital patients when the follow-up is greater than 6 months. |
| BMI  (low risk of bias) | MD -0.57 lower  [-1.20, 0.05] | 589 (5 RCTs) | ⨁◯◯◯ VERY LOW ^c,d,f^ | The evidence is very uncertain about the effect of behaviour change interventions on changes in BMI in ambulatory hospital patients when studies with a high risk of bias are excluded. |
|  |  |  |  |  |
| Waist Circumference  (<=6mo follow-up) | MD -3.91 lower  [-5.96, -1.85] | 194 (3 RCTs) | ⨁⨁◯◯ LOW ^c,d^ | The evidence suggests that behaviour change interventions results in a slight reduction in waist circumference in ambulatory hospital patients. |
| Waist Circumference  (>6mo follow-up) | MD -0.66 lower  [-2.28, 0.95] | 336 (2 RCTs) | ⨁◯◯◯ VERY LOW ^a,c,d^ | The evidence is very uncertain about the effect of behaviour change interventions on changes in waist circumference in ambulatory hospital patients. |
| Waist Circumference  (low risk of bias) | MD 2.34 lower  [-4.49, -0.18] | 472 (4 RCTs) | ⨁◯◯◯ VERY LOW ^c,d,f^ | The evidence is very uncertain about the effect of behaviour change interventions on changes in waist circumference in ambulatory hospital patients. |
|  |  |  |  |  |

***The risk in the intervention group** (and its 95% confidence interval) is based on the assumed risk in the comparison group and the **relative effect** of the intervention (and its 95% CI).

#### Explanations

a. Large number of studies with high risk of bias

b. High heterogeneity

c. Differences in population and outcome measures

d. Wide confidence intervals

e. Asymmetry in the pattern of results

f. Moderate heterogeneity
